# Supplementary material for: Multi-Omics Data Integration Reveals Key Variables Contributing to Subgingival Microbiome Dysbiosis-Induced Inflammatory Response in a Hyperglycemic Microenvironment
Source: Int J Mol Sci. 2023 May 16;24(10):8832. doi: 10.3390/ijms24108832 (PMC10218861; doi:10.3390/ijms24108832)
Supplement: Supplementary file 1 [file ijms-24-08832-s001.zip › ijms-2398711-supplementary.pdf]

Supplemental materials

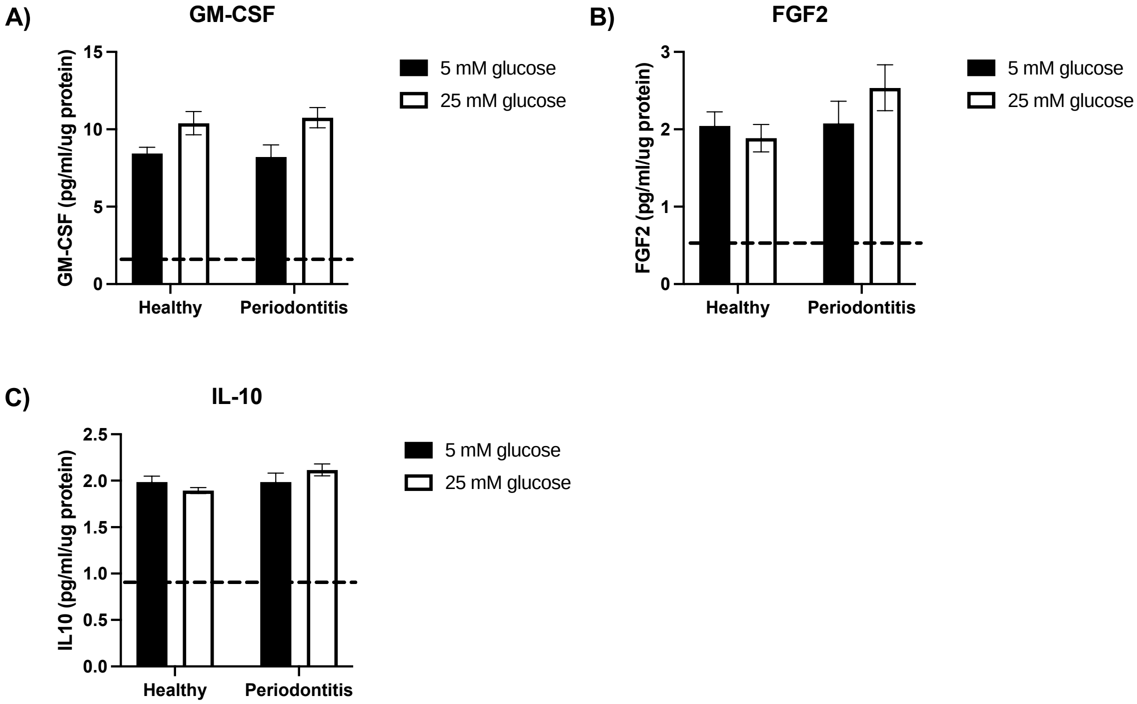

Figure S1. Pro-inflammatory cytokines and matrix metalloproteinases secretion by the gingival coculture model. Pro-inflammatory cytokines and matrix metalloproteinases secretion of **A)** GM-CSF, **B)** FGF2, and **C)** IL-10 by the coculture model following 24 h stimulation with subgingival microbiomes from healthy participants (healthy) and patients with periodontitis (periodontitis) in 5 mM and 25 mM glucose. The dash line represents the baseline secretion by the untreated coculture model in both 5 mM and 25 mM glucose. Results are the mean ± SEM. One independent replicate and in two technical replicates for each condition. Two-Way ANOVA with Tukey *post hoc* test.

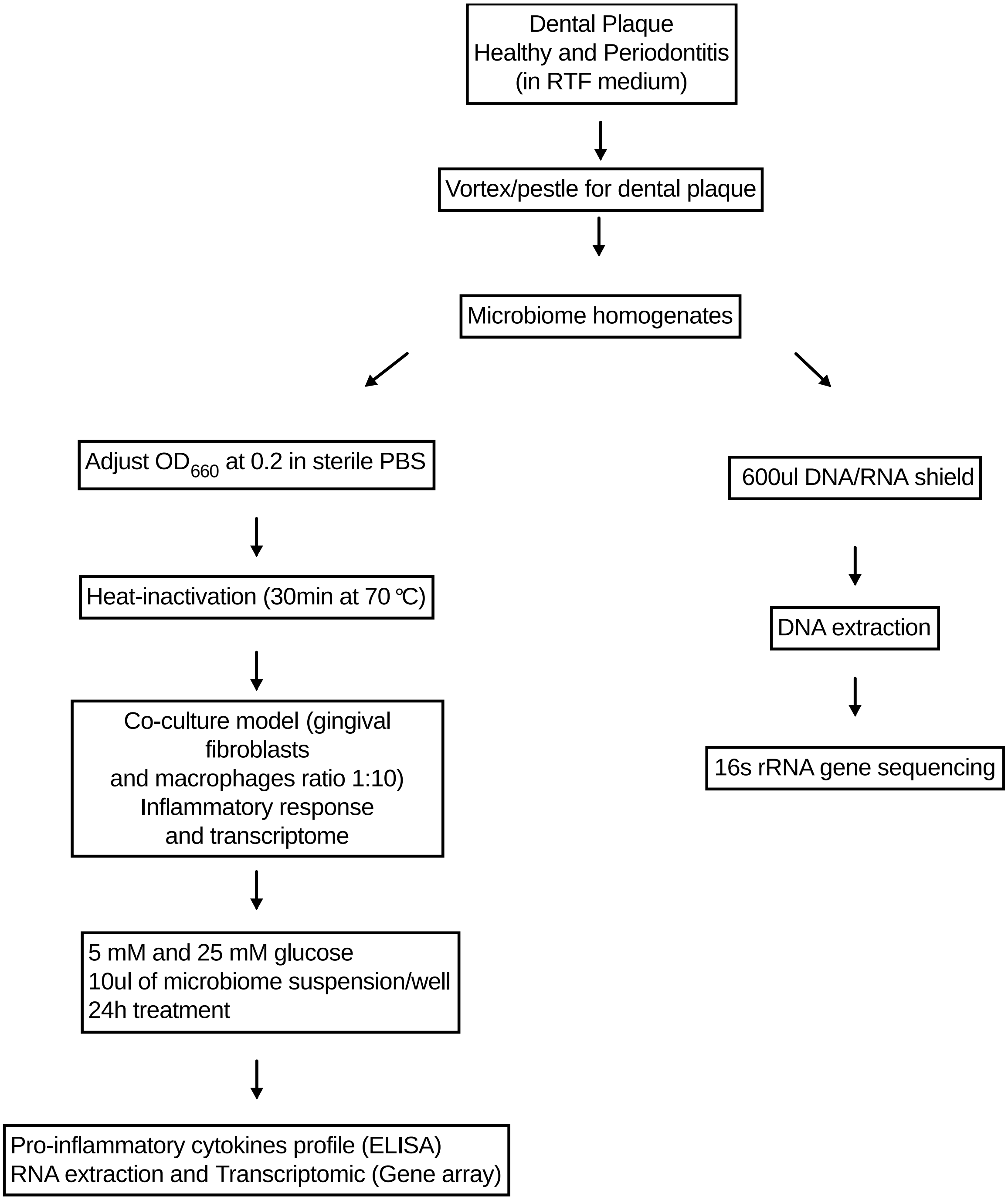

Figure S2. Experimental design.
